# The prognostic value of asymmetric dimethylarginine in patients with cardiac syndrome X

**DOI:** 10.1371/journal.pone.0188995

**Published:** 2017-12-05

**Authors:** Tse-Min Lu, Tzong-Shyuan Lee, Shing-Jong Lin, Wan-Leong Chan, Chiao-Po Hsu

**Affiliations:** 1 Division of Cardiology, Department of Internal Medicine, Taipei Veterans General Hospital, Taipei, Taiwan, R.O.C.; 2 Department of Health Care Center, Taipei Veterans General Hospital, Taipei, Taiwan, R.O.C.; 3 School of Medicine, National Yang-Ming University, Taipei, Taiwan, R.O.C.; 4 Department of Physiology, National Yang-Ming University, Taipei, Taiwan, R.O.C.; 5 Department of Medical Research and Education, Taipei Veterans General Hospital, Taipei, Taiwan, R.O.C.; 6 Division of Cardiovascular Surgery, Department of Surgery, Taipei Veterans General Hospital, Taipei, Taiwan, R.O.C.; Rutgers New Jersey Medical School, UNITED STATES

## Abstract

**Background:**

The pathophysiology of cardiac syndrome X is multifactorial and endothelial dysfunction has been implicated as important contributing factor. Asymmetric dimethylarginine (ADMA), characterized as a circulating endogenous inhibitor of nitric oxide synthase, may have been implicated as an important contributing factor for the development of endothelial dysfunction. In this study, we aim to assess the predictive power of ADMA for long-term prognosis in patients with cardiac syndrome X.

**Methods and results:**

We enrolled 239 consecutive patients with cardiac syndrome X diagnosed by coronary angiography. The mean age was 58.7±10.1 years. The patients were grouped into tertiles according to the plasma ADMA levels: <0.38 μmol/l (tertile I), 0.38–0.44 μmol/l (tertile II), and >0.44 μmol/l (tertile III). All patients were followed up for a mean period of 6.5±1.5 years (median: 6.3 years, inter-quartile range: 5.7–8.0 years). During the follow-up period, major adverse events (MAE) were observed in 15 patients (6.3%), including 13 deaths. The plasma ADMA levels in patients who developed MAE were significantly higher than those who did not (0.48±0.06 μmol/l vs. 0.42±0.08 μmol/l, p = 0.005). In multivariate Cox regression analysis adjusted for age, eGFR and LVEF, ADMA tertile I and II were identify to be associated with a significantly lower risk of MAE compared to ADMA tertile III (p = 0.017). By considering the plasma ADMA level as a continuous variable, the plasma ADMA level remained a significant independent predictor for outcomes of MAE, and the relative risk of MACE increased by 50% when plasma ADMA level increased by 1 SD of value (p = 0.018).

**Conclusions:**

In patients with cardiac syndrome X, elevated plasma ADMA levels appeared to be an independent predictor of long-term adverse clinical outcomes.

## Introduction

Cardiac syndrome X is a clinical entity which is defined by typical angina-like chest pain with normal or near-normal coronary angiography and exclusion of other causes of chest pain. [1, 2] These patients should have objective signs of ischemia, such as the classic down-sloping ST-segment depression on treadmill exercise test, and/or a reversible perfusion defect on radionuclear myocardial perfusion scan. The pathophysiology of cardiac syndrome X is multifactorial and endothelial dysfunction with subsequent microvascular ischemia has been implicated as an important contributing factor. [3, 4] Egashira et al. have showed that an acute intracoronary infusion of L-arginine might normalize nitric oxide (NO)-dependent coronary vasodilation in the patients with cardiac syndrome X, suggested that these patients might have a defect in NO bioavailability. [5] Therefore, as a well-characterized endogenous inhibitor of NO synthase, asymmetric dimethylarginine (ADMA) might be implicated as a potential contributing factor for the development of endothelial dysfunction as well as the pathogenesis of cardiac syndrome X. Plasma ADMA levels in patients with cardiac syndrome X have been shown to be significantly higher than those of normal subjects, and ADMA might be involved in the abnormal vascular reactivity that was always observed in these patients. [6] On the other hand, although patients with cardiac syndrome X always have been reported to have excellent long-term clinical prognosis, [7, 8] current evidences have suggested that a significant portion of these patients with endothelial dysfunction or microvascular dysfunction may be associated with higher risk of long-term adverse cardiovascular events. [9, 10] As the prognostic value of ADMA for cardiac syndrome X have never been addressed, in this study we aim to assess the predictive power of ADMA for future adverse cardiovascular events in our patients diagnosed as cardiac syndrome X after cardiac catheterization.

## Materials and methods

### Study population

From Jan 2007 to June 2009, we enrolled 239 consecutive patients scheduled to undergo coronary angiography for the evaluation of chest pain and/or suspected coronary artery disease and finally was diagnosed as cardiac syndrome X, which is defined as positive stress exercise test or radionuclear myocardial perfusion defect, and with normal or near-normal coronary artery at coronary angiography. Exclusion criteria included patients with severe liver disease and end-stage renal disease under regular hemodialysis, active infectious disease, chronic or acute inflammatory disease, malignancy with life expectancy less than 1 year, congestive heart failure, significant valvular heart disease and severe hypertension. Coronary blood flow was assessed by Thrombolysis In Myocardial Infarction (TIMI) frame count. Thorough medical histories of all participants were recorded. All medications, cigarette smoking and beverages containing alcohol or caffeine were withdrawn for at least 12 hours before blood draw. Fasting blood samples were collected in EDTA tubes for the measurement of ADMA, L-arginine, blood sugar, and for other biochemical analyses before the coronary angiography. All patients were prospectively followed by monthly office visit or by telephone contact and chart review for the occurrence of all-cause mortality and first-ever major adverse events (MAE), defined as all-cause mortality, stroke and non-fatal myocardial infarction. Myocardial infarction was defined as the presence of significant new Q waves in at least 2 electrocardiographic leads or of symptoms compatible with myocardial infarction associated with increase in creatine kinase-MB fraction ≥3 times the upper limit of the reference range. Stroke with neurological deficit was diagnosed by a neurologist on the basis of imaging study. The study protocol was approved by the Institutional Review Board at Taipei-Veterans General Hospital, and all participants provided written informed consent.

### Laboratory measurements

As described in detail previously [11], blood samples were centrifuged at 3000 rpm for 10 minutes promptly after collection and plasma was frozen until analysis. Plasma L-arginine and ADMA concentrations were determined by high performance liquid chromatography as earlier report. [12] The recovery rate for ADMA was more than 90%, and the within-assay and between-assay variation coefficients were not more than 7% and 8%, respectively. Fasting serum creatinine, total and high-density lipoprotein (HDL)-cholesterol, triglycerides, and blood sugar levels were determined by an auto-analyzer (Model 7600–310, Hitachi, Tokyo). Low-density lipoprotein (LDL)-cholesterol levels were computed according to the Friedewald formula. [13] The estimated glomerular filtration rate (eGFR) was calculated according to the simplified version of the Modification of Diet in Renal Disease Study prediction equation formula, modified by Ma et al. for Chinese patients (eGFR = 175 × plasma creatinine^-1.234^ × age^-0.179^ × 0.79 [if female]). [14]

### Statistical analysis

Continuous variables are expressed as the mean ± standard deviation or with confidence interval (CI) of 95%. Differences between groups were compared with one-way analysis of variance (ANOVA) or two-sample *t*-tests. Categorical variables were compared by Chi-square test or Fisher’s exact test. Pearson’s correlation coefficients were calculated to examine possible correlations between continuous variables. Actuarial event-free survival curves were estimated using the Kaplan-Meier method and compared by log-rank test. Receiver operating characteristic (ROC) analyses were used to compare the discrimination of the plasma ADMA level for the risk of MAE, and the predictive validities were quantified as the area under the ROC curves (*c*-statistic). The univariate Cox regression analysis was performed first to determine the association with the risk of MAE in all subjects. Those with a p value < 0.1 were included into the multivariate Cox regression analysis. The plasma ADMA level was tested as a continuous or as tertiles. The hazards ratio (HR) and 95% CI were calculated. A p value of less than 0.05 was considered statistically significant. The SPSS 17.0 (SPSS Inc., Chicago, Illinois) software package was used for statistical analysis.

## Results

### Baseline characteristics of the study population

The mean age of the 239 patients was 58.7 ± 10.1 years, and equal gender distribution (Male: female = 120: 119). Slow coronary flow (TIMI grade 2 or 1) was observed in 28 patients (11.7%). The mean plasma ADMA and L-arginine were 0.42 ± 0.08 μmol/l and 93.3 ± 32.7 μmol/l, respectively. The plasma ADMA levels in male and female patients and in slow/normal coronary flow were nearly identical (Male vs. Female: 0.42 ± 0.08 μmol/l versus 0.42 ± 0.08 μmol/l, p = 0.97; Slow coronary flow versus normal coronary flow: 0.42 ± 0.07 μmol/l versus 0.42 ± 0.09 μmol/l). Significant correlations were observed between plasma ADMA level and age (r = 0.26, p < 0.01) as well as eGFR (r = −0.15, p = 0.02), respectively. Patients were grouped into tertiles according to the plasma ADMA levels: < 0.38 μmol/l (tertile I), 0.38–0.44 μmol/l (tertile II), and >0.44 μmol/l (tertile III). The baseline clinical and angiographic characteristics of the study groups are summarized in Table 1. The patients in tertile III were older. In tertile III, there were more patients with hypertension and taking calcium channel blockers, anti-platelet agents and angiotensin converting enzyme/angiotensin receptor blockers. Furthermore, the creatinine level was higher and eGFR was lower in the tertile III (Table 1).

**Table 1:** Baseline characteristics.

	ADMA Tertile I < 0.38 μmol/l n = 79	ADMA Tertile II 0.38–0.44 μmol/l n = 80	ADMA Tertile III > 0.44 μmol/l n = 80	P value
Age (years)	55.3 ± 9.7	57.6 ± 10.5	63.3 ± 8.4	< 0.01
Gender (male, %)	40 (50.6%)	38 (47.5%)	42 (52.5%)	0.83
BMI (kg/m^2^)	25.7 ± 3.8	26.7 ± 4.2	27.2 ± 4.1	0.15
Hypertension (%)	47 (59.5%)	50 (62.5%)	65 (81.2%)	< 0.01
Diabetes (%)	16 (20.3%)	28 (35.0%)	17 (21.2%)	0.07
Hypercholesterolemia (%)	31 (39.2%)	35 (43.8%)	39 (48.8%)	0.49
Current smoker (%)	11 (13.9%)	15 (18.8%)	15 (18.8%)	0.65
Creatinine (mg/dl)	0.9 ± 0.3	0.9 ± 0.3	1.1 ± 0.4	0.01
Cholesterol (mg/dl)				
Total	184.6 ± 36.7	181.4 ± 31.8	176.7 ± 39.8	0.40
LDL	116.2 ± 33.5	111.6 ± 29.0	105.5 ± 34.6	0.13
HDL	47.9 ± 11.8	46.6 ± 12.5	48.4 ± 15.4	0.70
Triglyceride (mg/dl)	135.9 ± 73.3	150.9 ± 79.2	155.0 ± 112.8	0.38
Fasting blood sugar (mg/dl)	97.2 ± 24.3	110.3 ± 56.8	101.7 ± 38.9	0.59
eGFR (ml/min per 1.73 m^2^)	94.6 ± 30.4	92.0 ± 30.8	79.9 ± 29.3	<0.01
L-arginine (μmol/l)	86.7 ± 29.0	91.7 ± 35.0	101.2 ± 32.4	0.18
Slow coronary flow (%)	8 (10.1%)	12 (15.0%)	8 (10.0%)	0.56
LVEF (%)	60.9 ± 6.1	61.1 ± 7.8	59.0 ± 6.8	0.11
Medications				
Statins	27 (34.2%)	37 (46.2%)	41 (51.2%)	0.09
Calcium channel blocker	29 (36.7%)	45 (56.2%)	50 (62.5%)	<0.01
Anti-platelet agent	53 (67.1%)	59 (73.8%)	60 (75.0%)	0.02
Beta-blocker	40 (50.6%)	49 (61.2%)	37 (46.2%)	0.16
ACE inhibitor/ARB	21 (34.2%)	31 (38.8%)	44 (55.0%)	0.02

ACE: angiotensin-converting enzyme; ARB: angiotensin II receptor blocker; BMI: body mass index; eGFR: estimated glomerular filtration rate; LVEF: left ventricular ejection fraction

### Long-term outcome and ADMA

**Fig 1:**
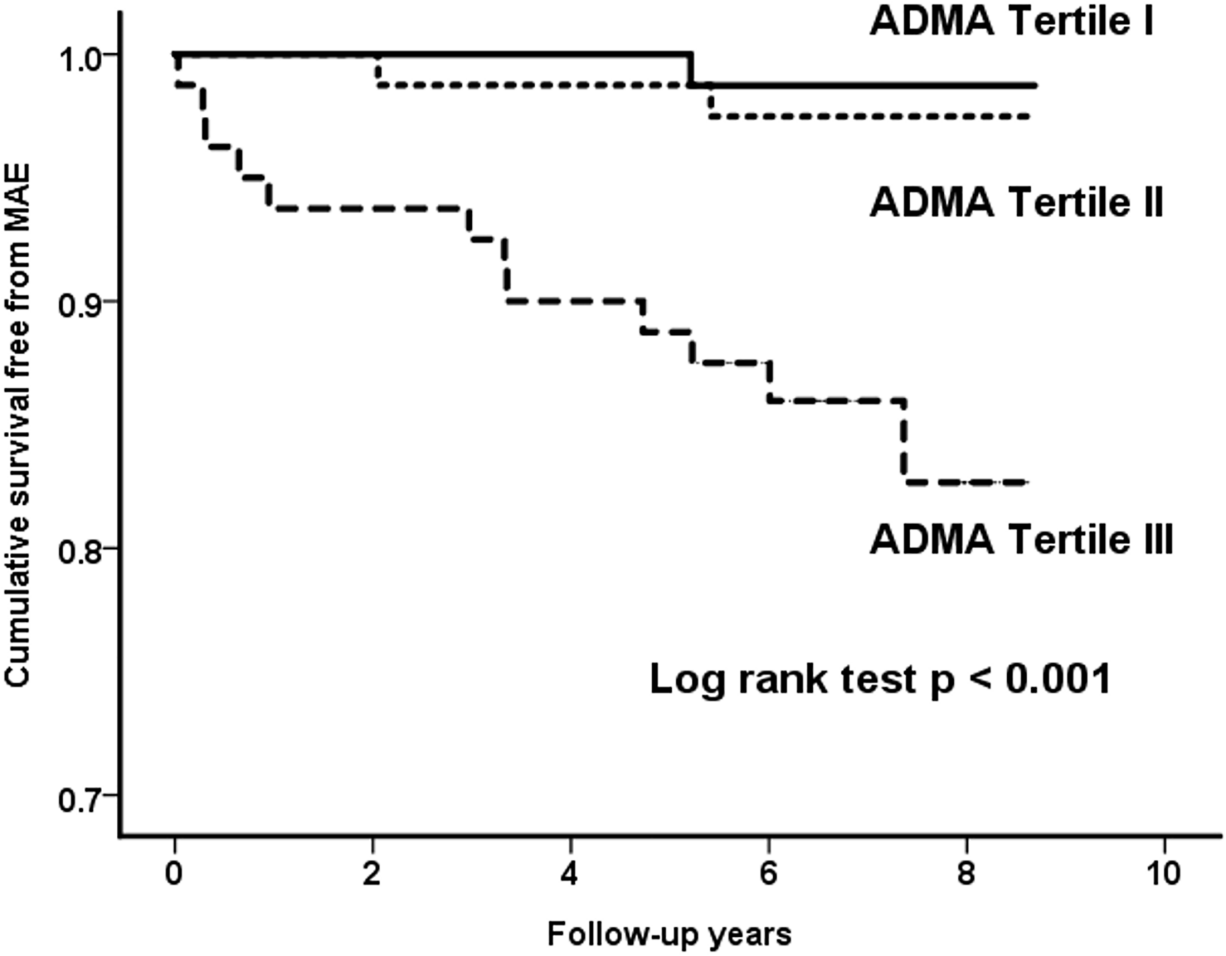
Cumulative survival curves free from major adverse events. Kaplan-Meier survival analyses during follow-up for major adverse events, according to the plasma ADMA tertiles. P values by log-rank test are shown.

All patients were followed up completely for a mean period of 6.5 ± 1.5 years (median: 6.3 years, inter-quartile range: 5.7–8.0 years) without any loss of follow-up. During the follow-up period, there were 13 patients (5.4%) died, including 8 cardiovascular deaths (3 fatal stroke). The other 4 patients died of cancer and the remaining 1 patient died of liver cirrhosis. MAE were observed in 15 patients (6.3%), with 2 additional cases of nonfatal MI. The plasma ADMA levels in patients who developed MAE were significantly higher than those who did not (0.48 ± 0.06 μmol/l vs. 0.42 ± 0.08 μmol/l, p = 0.005). Fig 1 showed the cumulative survival curves free from MAE determined using the Kaplan-Meier method in patients divided according to the plasma ADMA tertile, with the outcome being highly significantly worse in those patients of tertile III (p < 0.001). In particular, only 1 and 2 MAE occurred in patients of tertile I and II respectively, compared to 12 events in patients of tertile III. Therefore, in multivariate Cox regression analysis, we grouped the tertile I and II together and compared their outcomes with those of tertile III. After adjusted for age and eGFR and left ventricular ejection fraction (LVEF), ADMA tertile I and II were identify to be associated with a significantly lower risk of MAE compared to ADMA tertile III (HR: 0.12, 95% CI: 0.05–0.75, p = 0.017, Table 2). By considering the plasma ADMA level as a continuous variable, the plasma ADMA level remained a significant independent predictor for risk of MAE, and the relative risk of MAE increased by 50% when plasma ADMA level increased by 1 standard deviation of value (p = 0.018, Table 2). By ROC curve analysis, the areas under the curve (*c*-statistics) of plasma ADMA level for the occurrence of MAE were 0.769 (95% CI: 0.642–0.896, p<0.001). By using a plasma ADMA level of 0.47 μmol/l as the cutpoint, the analysis yielded 66.7% sensitivity, 83.5% specificity, 21.3% positive predictive rate and 97.4% negative predictive rate.

**Table 2:** Univariate and multivariate Cox regression analysis for MAE.

	*Univariate analysis*		*Multivariate analysis*	
	HR (95% CI)	P	HR (95% CI)	P
Age (years) (/increase of 1 SD)	2.17 (1.17–4.01)	0.014	1.77 (0.94–3.35)	0.078
eGFR (/increase of 1 SD)	0.41 (0.21–0.81)	0.010	0.50 (0.24 − 1.03)	0.059
DM	0.72 (0.20–2.75)	0.611		
Current smoker	2.33 (0.80–6.81)	0.123		
Hypertension	0.94 (0.32–2.75)	0.909		
Slow coronary flow	0.52 (0.15–1.83)	0.307		
LVEF (/increase of 1 SD)	0.58 (0.34–0.99)	0.044	0.62 (0.36 − 1.07)	0.087
Use of CCB	1.85 (0.63–5.43)	0.260		
ADMA				
Continuous (/1 SD increase of ADMA)	1.53 (1.14–2.07)	0.005	1.47 (1.04–2.09)	0.030
ADMA Tertile				
I versus III	0.08 (0.01–0.64)	0.017	0.14 (0.02–1.13)	0.065
II versus III	0.16 (0.04–0.71)	0.016	0.23 (0.05–1.04)	0.056
I plus II versus III	0.12 (0.03–0.43)	0.001	0.19 (0.05–0.70)	0.013

CCB: Calcium channel blocker; CI: confidence interval; eGFR: estimated glomerular filtration rate; HR: hazard ratio; LVEF: left ventricular ejection fraction, SD: standard deviation.

All the data could be found and analyzed in the S1 File.

## Discussions

The results of this study suggested that plasma ADMA levels were significantly associated with long-term adverse events in patients with cardiac syndrome X. Our findings showed the value of measuring ADMA levels for predicting adverse events in these patients with positive evidence of ischemia but with normal coronary angiogram.

### Microvascular dysfunction, cardiac syndrome X and ADMA

It has been noted that cardiac syndrome X may be associated with microvascular dysfunction, which is caused by a variable combination of impaired endothelium-independent and endothelium-dependent coronary vasodilation. [4, 15] As a circulating endogenous inhibitor of NO synthase, ADMA may compete with L-arginine as the substrate for NO synthase, and may decrease the production of NO. [16, 17] Elevated ADMA level might also inhibit the mobilization, differentiation and function of endothelial progenitor cell. [18] In addition, ADMA may increase oxidative stress both by angiotensin-II- NADPH pathway and by uncoupling the electron transport between NO synthase and L-arginine, which can lead to the decrease in the production and availability of endothelium-derived NO. [19, 20] Therefore, ADMA has been implicated as one of the contributing factors for the pathogenesis of endothelial dysfunction. Previous studies have showed that elevations of plasma ADMA levels might be associated with the presence of endothelial dysfunction, [17] and has been observed in patients with various risk factors of atherosclerosis. [21–23] In contrast, the relation between ADMA and cardiac syndrome X have less been addressed. The plasma levels of ADMA in patients with cardiac syndrome X were reported to be higher than those of the control group, and increased plasma ADMA levels were associated with impaired myocardial tissue perfusion assessed by myocardial blush grade. [6] Sen et al. showed that the plasma level of nitrate/nitrate, L-arginine, and L-arginine/ADMA ratio were lower in patients with cardiac syndrome X than they were in the control group subjects, and the plasma ADMA levels were positively correlated with carotid intima media thickness in patients with cardiac syndrome X. [24] Furthermore, Piatti et al. also reported that plasma ADMA levels are increased in patients with cardiac syndrome X, and they correlated with the increases in endothelin-1 and reductions in insulin-induced increments in plasma nitrite/nitrate and cGMP, effects that are reversed by intravenous L-arginine infusion. [25] All of these evidences suggested that increased ADMA levels might play a role in the endothelial dysfunction observed in patients with cardiac syndrome X.

### The long-term prognosis of cardiac syndrome X and ADMA

Current evidence suggested cardiac syndrome X is associated with long-term adverse events, including MI, stroke, heart failure, and death. [9, 10] In contrast, endothelial dysfunction has been reported to be associated with long term adverse events in patients with varying severity of coronary artery disease, including cardiac syndrome X, [7, 9] whereas the ADMA has also been reported to be an independent risk factor for patients with coronary artery disease. [12, 26–28] Regarding the close association between the pathogenesis of endothelial dysfunction and reduced bioavailability of nitric oxide, ADMA might play some role in predicting the long-term adverse events in patients with cardiac syndrome X, but their relation has never been reported. To the best of our knowledge, our study is the first one to show that plasma ADMA levels might predict the long-term adverse events of patients with cardiac syndrome X. Although the long-term prognosis of our patients with cardiac syndrome X appeared to be good, with only 15 events (6.3%) during the follow-up, these results were comparable to those previously reported, [8] and the plasma ADMA levels remained a significant predictor for long-term adverse events even in the multi-variable Cox regression model adjusted for age, smoker, hypertension, diabetes and renal function. Nevertheless, our results needed to be confirmed in larger studies.

### Limitations

Several limitations of this study need to be addressed. First, this study was a single-center observational study with small sample size, and therefore, fewer adverse events were observed. With regard to the endpoints of MAE, the estimated study power of this study would be 0.86, and confirmation of our findings in a cohort involving more patients with cardiac syndrome X is needed. Second, we did not measure the endothelium-dependent coronary vasodilation in our patients with cardiac syndrome X. Direct measurement of endothelium-dependent coronary vasodilation, like by intracoronary acetylcholine infusion test, may be informative in elucidating the relation of ADMA and cardiac syndrome X.

## Conclusions

In patients with cardiac syndrome X, elevated plasma ADMA levels appeared to be an independent predictor of long-term adverse clinical outcomes.

## Supporting information

S1 File (SAV)
